# Using Longitudinal Twitter Data for Digital Epidemiology of Childhood Health Outcomes: An Annotated Data Set and Deep Neural Network Classifiers

**DOI:** 10.2196/50652

**Published:** 2024-03-25

**Authors:** Ari Z Klein, José Agustín Gutiérrez Gómez, Lisa D Levine, Graciela Gonzalez-Hernandez

**Affiliations:** 1 Department of Biostatistics, Epidemiology, and Informatics Perelman School of Medicine University of Pennsylvania Philadelphia, PA United States; 2 Department of Health Sciences University of Monterrey San Pedro Garza García, Nuevo León Mexico; 3 Department of Obstetrics and Gynecology Perelman School of Medicine University of Pennsylvania Philadelphia, PA United States; 4 Department of Computational Biomedicine Cedars-Sinai Medical Center West Hollywood, CA United States

**Keywords:** natural language processing, machine learning, data mining, social media, Twitter, pregnancy, epidemiology, developmental disabilities, asthma

## Abstract

We manually annotated 9734 tweets that were posted by users who reported their pregnancy on Twitter, and used them to train, evaluate, and deploy deep neural network classifiers (*F*_1_-score=0.93) to detect tweets that report having a child with attention-deficit/hyperactivity disorder (678 users), autism spectrum disorders (1744 users), delayed speech (902 users), or asthma (1255 users), demonstrating the potential of Twitter as a complementary resource for assessing associations between pregnancy exposures and childhood health outcomes on a large scale.

## Introduction

Many children are diagnosed with disorders that can impact their daily lives and can last throughout their lifetime. For example, in the United States, 17% of children are diagnosed with a developmental disability [1] and 8% of them with asthma [2]. Meanwhile, data sources for assessing the association of these outcomes with pregnancy exposures are limited, as pregnancy registries typically follow infants for up to 1 year after birth. While our previous work [3,4] demonstrated the utility of Twitter as a source of data regarding pregnancy outcomes, the ability to continue collecting users’ tweets on an ongoing basis after birth may present opportunities to detect outcomes in childhood. Twitter data have been used to identify self-reports of attention-deficit/hyperactivity disorder (ADHD) [5], autism spectrum disorders (ASD) [6], and asthma [7], but not to identify reports of these disorders in users’ children. This study aimed to assess whether there are users who report having a child with ADHD, ASD, delayed speech, or asthma, and develop and evaluate an automated method for identifying these reports.

## Methods

### Ethical Considerations

The study data were collected and analyzed in accordance with the Twitter Terms of Service. The institutional review boards of the University of Pennsylvania and Cedars-Sinai Medical Center deemed this study exempt.

### Data Collection

We searched for mentions of ADHD, ASD, delayed speech, and asthma among all the tweets posted by more than 100,000 users who reported their pregnancy on Twitter [8]. We then searched these matching tweets for references to a child and the user, and excluded tweets that matched specific patterns indicating the user’s own disorder. The query (Multimedia Appendix 1) returned 36,094 tweets (excluding retweets) posted by 11,712 users.

### Annotation

We used 400 matching tweets—100 per outcome—to develop annotation guidelines (Multimedia Appendix 2) for distinguishing those that report having a child with a disorder from those that do not. An additional 9334 tweets—1 random tweet per user—were then independently annotated: 8334 by 2 annotators and 1000 by all 3. Interannotator agreement (Fleiss kappa) was 0.88. After resolving disagreements among all 9734 tweets, we determined that 3019 (31%) reported having a child with a disorder and 6715 (69%) did not.

### Automatic Classification

We split the 9734 tweets into 80% (n=7787) training (Multimedia Appendix 3) and 20% (n=1947) test data, and performed machine learning experiments using deep neural network classifiers based on bidirectional encoder representations from transformers (BERT) [9]: the BERT-Base-Uncased, RoBERTa-Large, and BERTweet-Large pretrained models in the *Huggingface* library. Our preprocessing included normalizing URLs and usernames, and lowercasing the tweets. For training, we used Adam optimization, 5 epochs, a batch size of 8, and a learning rate of 0.00001, based on evaluating after each epoch using a 5% split of the training set. We fine-tuned all layers of the models with our annotated tweets.

## Results

Table 1 presents the performance of the classifiers. The RoBERTa-Large [10] classifier achieved the highest overall *F*_1_-score (0.93). Table 1 also presents the performance of the RoBERTa-Large classifier for tweets that mention specific outcomes. We deployed the RoBERTa-Large classifier on the additional 26,360 unlabeled tweets that matched our query (Multimedia Appendix 1). Between the 9734 manually annotated tweets and the 26,360 automatically classified tweets, we identified 3806 total users who reported having a child with ADHD (n=678), ASD (n=1744), delayed speech (n=902), or asthma (n=1255).

Table 2 presents examples of tweets in the test set that were misclassified by the RoBERTa-Large classifier. While 28 (58%) of the 48 false positives do refer to the user’s child, 11 (39%) indicate that someone other than the user’s child has a disorder (tweet 1), and 9 (32%) indicate that a disorder is merely suspected or exhibited (tweet 2). Among the other 20 (42%) of the 48 false positives, 10 (50%) are reported speech, such as quotations (tweet 3). Among the 42 false negatives, 22 (52%) do not explicitly mention the user’s child (tweet 4)—for example, using a pronoun or name—and 14 (33%) do not explicitly indicate that the child has a disorder (tweet 5).

**Table 1:** Precision, recall, and *F*_1_-score of classifiers for the class of tweets that report having a child with attention-deficit/hyperactivity disorder (ADHD), autism spectrum disorder (ASD), delayed speech, or asthma, including the outcome-specific precision, recall, and *F*_1_-score for the RoBERTa-Large classifier.

Classifier		Precision	Recall	*F*_1_-score
BERT-Base-Uncased		0.83	0.87	0.85
BERTweet-Large		0.89	0.94	0.92
**RoBERTa-Large**		0.92	0.94	0.93
	ADHD	0.91	0.85	0.88
	ASD	0.94	0.92	0.93
	Delayed speech	0.94	0.96	0.95
	Asthma	0.91	0.96	0.94

**Table 2:** Sample false positives and false negatives of a RoBERTa-Large classifier for detecting tweets that report having a child with attention-deficit/hyperactivity disorder (ADHD), autism spectrum disorder (ASD), delayed speech, or asthma (with the text that matched the data collection query in italics).

Tweet number	Tweet	Actual	Predicted
1	So Maxine Waters can be maskless on a plane but *I* can’t fly with *my 2 year old* cause she won’t wear a mask? *Kids* with *autism* are being banned from flying because they won’t wear a mask?	–	+
2	they treat *my baby* with *asthma* meds all the time but didn’t diagnose her with it im pretty sure she has it tho	–	+
3	Any tips for this mum: “*My daughter* is 10. *My* parents would like to gift her either a phone or a smart watch which is easy to use and won’t be easily damaged by a very active *ADHD kid*... *I* need help choosi… [URL]	–	+
4	Flying tomorrow...during a pandemic with a *nonverbal 3 year old*. *We* could use some prayers, please. 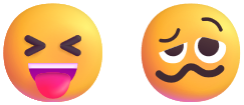	+	–
5	*I* wouldn’t change *my child* for anything in the world. *I*’m just curious to know where *autism* came from because *me* and his dad don’t have any family members that are *autistic*. It’s just a question out of curiosity	+	–

## Discussion

Our ability to identify Twitter data during pregnancy for users who reported having a child with ADHD, ASD, delayed speech, or asthma suggests that Twitter could be a complementary resource for assessing associations between pregnancy exposures and childhood health outcomes, with potential clinical implications for informing prenatal care. The overall and outcome-specific performance for automatically identifying these outcomes demonstrates the feasibility of using Twitter data for observational studies on a large scale.

## Appendix

Multimedia Appendix 1 Data collection query.

Multimedia Appendix 2 Annotation guidelines.

Multimedia Appendix 3 Training data.

## Abbreviations

ADHD: attention-deficit/hyperactivity disorder
ASD: autism spectrum disorder
BERT: bidirectional encoder representations from transformers

## Data Availability

The manually annotated training data are included with this article in Multimedia Appendix 3. In accordance with the Twitter Terms of Service, these tweets are made available as tweet IDs, which can be rehydrated as tweet objects if they remain public at the time they are requested through the Twitter API.
