# Supplementary material for: Using Longitudinal Twitter Data for Digital Epidemiology of Childhood Health Outcomes: An Annotated Data Set and Deep Neural Network Classifiers
Source: J Med Internet Res. 2024 Mar 25;26:e50652. doi: 10.2196/50652 (PMC11002733; doi:10.2196/50652)
Supplement: Multimedia Appendix 2 [file jmir_v26i1e50652_app2.docx]

**Identifying Reports of Childhood Disorders on Twitter:**

**Annotation Guidelines**

The following brief guidelines will help annotators distinguish between tweets that do or do not report having a child who has asthma, attention-deficit/hyperactivity disorder (ADHD), autism spectrum disorders (ASD), delayed speech, or is otherwise non-verbal. Annotators should label tweets based only on the information provided directly in the tweet.

Tweets should be labeled as “1” if they indicate that the user’s child has any of the above medical conditions, as in:

1. Finally a dr has diagnosed my 3.5yr old with asthma. Now he will be on chronic medicine and we can hopefully keep him healthy and thriving. I'm over ER visits at 2am and setting alarms to go check if he is breathing. With a diagnosis you can plan and prevent. Best xmas gift.

While [1] indicates that the user’s child is 3.5 years old, [2] indicates that the child is much older:

1. It’s 4;53 and I’m still up.. my 20 year old is still up. He is concerned he has the Corona Virus because my daughter in love and granddaughters visited from Western WA yesterday. He is on the Autism Spectrum so it doesn’t have to be reasonable. High IQ HIGH emotions

Although the user who posted [2] may be excluded from particular research applications based on the age of the child, annotators should disregard the age and label [2] as “1” for the purpose of this annotation task.

Similarly, users may be excluded from particular research applications if they report that their child’s speech delay was related to another medical condition (e.g., hearing problems), but tweets such as [3] should also be labeled as “1” for the purpose of this annotation task:

1. i think if your kid is speech delayed u should get their ears done before jumping the gun cause cam picked up 4 new words literally within a week of it…. sometimes they really just can’t hear 🥴

While [3] explicitly refers to a speech delay, [4] merely indicates that the user’s child was non-verbal:

1. Henry went from non-verbal to cute babbles to SCREECHING/YELLING NON STOP so quickly While his happy screams are very cute, it is just very loud constantly in my ear holes. Babies, ya kno 🤷🏻‍♀️

Annotators should distinguish between when *non-verbal* does and does not refer to a speech delay. In [4], *non-verbal* seems to refer a normal developmental stage, so it should be labeled as “0”. Nonetheless, annotators may assume that the referent of *Henry* in [4] is the user’s child.

Tweets should also be labeled as “0” if they indicate that the condition is suspected but not diagnosed, as in:

1. @7menza @ZuvaSeven Can u give any tips to “live with it” please. I think my son has ADD. Trying to help him

In general, tweets should be labeled as “0” if they quote what was said by others, as in:

1. Anna from Wisconsin says, “My 11-year-old son has asked for prayer. Since getting a dog a few weeks back, he has developed severe asthma.”
2. My Daughter With ADHD Started Using a Weighted Blanket, and I Saw a Change in Her Almost Immediately <https://t.co/X8YyALeTsC>
3. We're grateful for your post! RT @_natalienya: RT @Dottheisllc: I don't usually post my family but... yesterday our non verbal son with autism celebrated his 29th birthday!

In [6], the report of asthma is wrapped in quotation marks and attributed to *Anna*. In [7], the report of ADHD appears to be in the title of a linked article being shared by the user. In [8], the report of autism is in a retweet.
